# Improved Diagnosis of Adjacent Organ Invasion of Extrahepatic Cholangiocarcinoma by Adding Arterial and Delayed Phases

**DOI:** 10.7759/cureus.47568

**Published:** 2023-10-24

**Authors:** Eisuke Mukaida, Akio Tamura, Kenichi Kato, Yoshitaka Ota, Satoshi Kasugai, Hirokatsu Katagiri, Hiroyuki Nitta, Nishiya Masao, Naoki Yanagawa, Kunihiro Yoshioka

**Affiliations:** 1 Department of Radiology, Iwate Medical University School of Medicine, Morioka, JPN; 2 Center for Radiological Science, Iwate Medical University Hospital, Morioka, JPN; 3 Department of Internal Medicine, Division of Gastroenterology, Iwate Medical University School of Medicine, Morioka, JPN; 4 Department of Surgery, Iwate Medical University School of Medicine, Morioka, JPN; 5 Department of Molecular Diagnostic Pathology, Iwate Medical University School of Medicine, Morioka, JPN

**Keywords:** computed tomography (ct), arterial and delayed phases, extrahepatic cholangiocarcinoma, adjacent organ invasion, improved diagnosis

## Abstract

Purpose: To clarify the role of dynamic computed tomography (CT) in diagnosing extrahepatic cholangiocarcinoma (eCCA) involving adjacent organs.

Material and methods: We retrospectively analyzed patients diagnosed with eCCA in Iwate Medical University Hospital (Morioka, Japan) during January 2011-December 2021 who underwent dynamic contrast-enhanced CT before biliary intervention, surgery, or chemotherapy. For surgical cases, two radiologists independently reviewed CT images in the portal, dual (adding arterial phase), and triple (adding delayed phase) phases. The mean attenuations of the abdominal aorta, portal vein (PV), hepatic parenchyma, pancreatic parenchyma, and eCCA were measured. The biliary segment-wise longitudinal tumour extent, arterial and PV invasion, organ invasion (liver, pancreas, and duodenum), and regional lymph node metastasis were assessed on a five-point scale. Image performances were compared using the sensitivity, specificity, and area under the curve (AUC).

Results: We included 120 patients (mean age, 71.7 ± 8.9; 84 males). The PV and liver differed most from the bile duct tumour in the portal phase. The abdominal aorta and pancreas differed most from eCCA in the arterial phase. For 80 patients evaluated on the five-point scale, adding phases increased the AUC for pancreatic, duodenal, and arterial invasion for each observer (observer 1, 0.79-0.93, p<0.01, 0.71-0.86, p = 0.04, 0.74-0.99, p = 0.02; observer 2, 0.88-0.96, p = 0.01, 0.73-0.94, p<0.01, 0.80-0.99 p = 0.04; respectively). The AUC for biliary segment-wise longitudinal tumor extent, hepatic, and PV invasion remained unchanged with additional phases.

Conclusions: Portal-phase information is sufficient to evaluate the segmental extent of bile duct and liver/PV invasion. Arterial- and delayed-phase information can help evaluate pancreatic, duodenal, and arterial invasion.

## Introduction

Cholangiocarcinoma (CCA) is a rare primary malignant epithelial neoplasm originating in the bile duct [1]. Its global incidence ranges from 0.3 to six cases per 100,000 inhabitants annually, with a mortality rate of one to six cases per 100,000 inhabitants per year [2]. East Asia has particularly high incidence rates of CCA [2]. CCA is categorized into two types: intrahepatic CCA (iCCA), which develops within the intrahepatic biliary tree and accounts for 10-20% of cases, and extrahepatic CCA (eCCA), which occurs outside the liver parenchyma. eCCA includes perihilar CCA (pCCA, 50% of cases) and distal CCA (dCCA, 30-40% of cases) [3]. Surgical resection is the only potentially curative option for CCA; however, the five-year survival rate after resection ranges from 25% to 40% [3]. Factors associated with low survival rates include vascular invasion, organ invasion, and residual tumor lesions after resection [4-6].

Multidetector computed tomography (MDCT) is commonly used to determine the surgical indication and operative procedure for eCCA owing to its ability to provide high-quality images of the intrahepatic and extrahepatic biliary systems, as well as the location of eCCA and surrounding organ involvement [7]. MDCT allows for visualizing contrast-enhanced peaks in various structures, including the liver, pancreas, liver arteries, and portal vein, which are adjacent to eCCA. Dynamic computed tomography (CT) through multiphase imaging captures these contrast-enhanced peaks. The National Comprehensive Cancer Network Clinical Practice Guidelines recommend multiphase MDCT as a preoperative examination [8]. However, unlike the guidelines for pancreatic adenocarcinoma, these guidelines do not specify the CT protocol, section thickness, scan acquisition timing, CT image reconstruction thickness, or image evaluation method [8,9]. This is because a standardized image evaluation method has not yet been established. Furthermore, the diagnostic performance of eCCA may be affected by the contrast effect of adjacent organs, which varies depending on the site of origin of eCCA.

The objective of this study is to determine the optimal CT imaging phase for assessing various aspects of eCCA.

## Materials and methods

Patients

This study adhered to the Declaration of Helsinki (revised in 2013). The study design received approved from Iwate Medical University Ethics Review Board (approval no. MH2022-084), and the requirement for informed consent was waived due to the study’s retrospective nature.

Data collection

One author (E.M.) searched Iwate Medical University Hospital (Morioka, Japan) radiology information system (RIS) for CT examinations conducted between January 2011 and December 2021 using the descriptive term “cholangiocarcinoma.” This study’s inclusion criteria were as follows: (a) age ≥18 years at the time of diagnosis; (b) undergoing dynamic-enhanced CT, including arterial-phase, portal-phase, and delayed-phase imaging with a section thickness of ≤3 mm at our tertiary referral hospital; and (c) confirmed eCCA based on pathological examination through surgical resection or needle biopsy. The exclusion criteria were as follows: (a) unestablished clinical diagnosis of eCCA; (b) prior biliary intervention, surgery, or chemotherapy before CT; (c) absence of CT protocol information; and (d) renal dysfunction (estimated glomerular filtration rate (eGFR) <45 mL/min/1.73 m²). All included patients underwent baseline CT within four weeks of the histologic diagnosis before any procedures, such as biopsy or surgery.

CT protocol

All patients underwent multiphasic CT using various types of MDCT scanners, including Aquilion CXL 64-row (Canon Medical Systems, Tochigi, Japan), Aquilion One 320-row (Canon Medical Systems), and LightSpeed VCT 64-row (GE Health Care, Milwaukee, Wisconsin, USA) with 120 kVp tube voltage and automatic exposure control. The section thickness of the images in all phases was ≤3 mm. Nonionic contrast medium containing 300 mg/mL iodine was administered through the antecubital vein via a 20-gauge catheter at 600 mgI/kg using a power injector (Nemoto DUAL SHOT Type D, Tokyo, Japan) with a fixed injection duration of 30 s. The scan delays were determined using an automatic bolus-tracking program. Arterial-phase imaging was automatically triggered 20 seconds after the abdominal aorta reached a threshold of 100 Hounsfield units (HU). This was followed by portal phase imaging 45 seconds later and delayed phase imaging 180 seconds later.

Objective assessment of CT images

For each patient, a medical student (C.S., third-year student) under the supervision of a radiologist (E.M., with four years of experience in abdominal CT) measured the mean attenuation of the abdominal aorta, portal vein, hepatic parenchyma (anterior, posterior, and lateral segments), and pancreatic parenchyma (head and body of the pancreas). Circular regions of interest (ROIs) were placed on one slice for the abdominal aorta (100 mm² ± 10 mm²), and the ROIs for the portal vein (extrahepatic portal vein) and hepatic parenchyma were set at 30 mm² ± 5 mm². The ROI for the pancreatic parenchyma was set at 20 mm² ± 5 mm². A radiologist (E.M.) measured the mean attenuation of the bile duct tumor using hand-drawn ROIs on three slices.

Subjective assessment of CT images

Subjective assessment was conducted on patients who underwent surgery for CCA and met the inclusion criteria. Two blinded radiologists (E.M. and A.T., with four and 14 years of experience in abdominal CT, respectively) independently evaluated CT images at the portal phase, dual phase (adding arterial phase), and triple phase (adding delayed phase), with a four-week interval between sessions. Standard abdominal window settings (width, 300 HU; level, 50 HU) were used to view the images. The radiologists evaluated the quality of axial CT images to determine the presence of tumors in the bile duct, as well as organ invasion (liver, pancreas, and duodenum) and vascular involvement. They used a five-point scale for grading with the following categories. For the presence of tumors in the bile duct, they assessed 1 for the tumor's form and contrast enhancement that were not recognizable; 2 for visible thickening of the bile duct wall, but not significant; 3 for unclear contrast enhancement of the tumor or contrast with surrounding organs, with indirect findings; 4 for visible contrast enhancement of the tumor or contrast with surrounding organs; and 5 for strong contrast enhancement of the tumor or unmistakably clear contrast with surrounding organs. In the case of organ invasion, the radiologists evaluated 1 for no contact between the bile duct tumor and the organ; 2 for minimal contact between the bile duct tumor and the organ, but not significant; 3 for minimal contact between the bile duct tumor and the organ, with unclear contrast enhancement of the invasion lesion or contrast with the organ; 4 for the invasion lesion exhibited contrast enhancement or contrast with the organ; and 5 for the invasion lesion displayed strong contrast enhancement or a clear contrast with the organ.

For a subjective assessment of vascular invasion, the radiologists targeted specific structures, including the celiac artery, superior mesenteric artery, common hepatic artery (right and left hepatic artery), and portal vein (extrahepatic portal vein-superior mesenteric vein). They used a five-point scale to determine: 1 for no contact between the bile duct tumor and the vascular; 2 for minimal contact between the bile duct tumor and the vascular (tumor covering ≤50% of vessel circumference), with no vessel deformity, occlusion, or tumor thrombus; 3 for minimal contact between the bile duct tumor and the vascular (tumor covering ≤50% of vessel circumference), but with visible vessel deformity, occlusion, or tumor thrombus; 4 for the bile duct tumor covered more than 50% of the vascular; and 5 for the bile duct tumor covered more than 50% of the vascular, with visible vessel deformity, occlusion, or tumor thrombus. The assessment of lymph node metastasis followed a proprietary five-point scale: 1 for undetected lymph nodes; 2 for detected lymph nodes with a maximal short-axis diameter less than 10 mm; 3 for lymph nodes with a diameter greater than 10 mm but less than 15 mm; 4 for maximal short-axis diameter exceeding 15 mm; and 5 for lymph nodes larger than 15 mm with tension and internal necrosis. The CT findings were compared with the findings of surgical and final pathological reports.

Statistical analysis

The mean attenuations of bile duct tumors and abdominal organs in each ROI are reported as mean CT values (HU) ± standard deviation (SD). Differences in CT values were evaluated using Welch’s analysis of variance (ANOVA) followed by the Bonferroni adjustment. The sensitivity, specificity, and AUC were also evaluated to compare each reader’s score to the pathological findings. DeLong's test was used to determine statistical significance for two correlated ROC curves with a p-value less than 0.05 To assess interobserver variability at interpreting images, kappa statistics were used to measure the degree of agreement. A kappa value of up to 0.20 stood for slight agreement, 0.21-0.40 for fair agreement, 0.41-0.60 for moderate agreement, 0.61-0.80 for substantial agreement, and 0.81 or greater for almost perfect agreement. All statistical analyses were performed using R software (version 4.3.1).

## Results

Patient demographics

**Figure 1:**
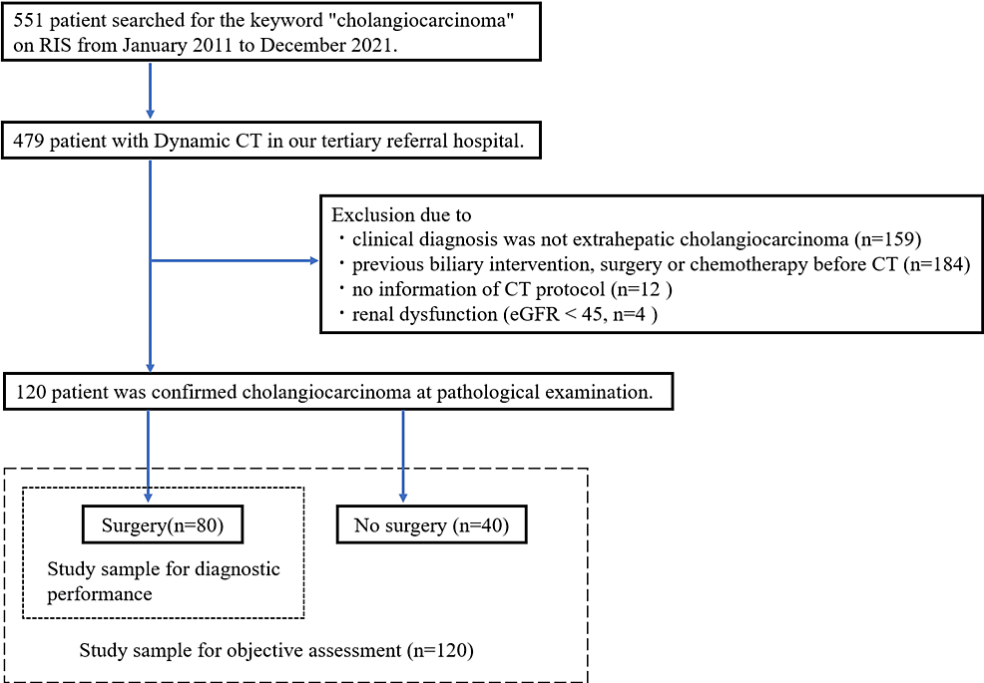
Flow diagram of the study sample. RIS, radiology information system; CT, computed tomography; eGFR, estimated glomerular filtration rate

The RIS search resulted in 551 patients with “cholangiocarcinoma”. Among those patients, 479 underwent dynamic CT at our tertiary referral hospital. A total of 159 patients were excluded because they were not diagnosed with eCCA, and 184 patients were excluded because they underwent previous biliary intervention, surgery, or chemotherapy before CT. Twelve patients were excluded because of missing information on the CT protocol. Four patients were excluded due to renal dysfunction (eGFR <45 mL/min/1.73 m²). Accordingly, 120 patients comprised the final study sample (Fig. 1 and Table 1).

**Table 1:** Patient characteristics In parentheses, percentages are shown. PV, portal vein

Characteristic	Value
Mean age (years)	71.7±8.9
Sex	
Male	84 (70.0)
Female	36 (30.0)
Histologic confirmation methods of extrahepatic bile duct cancer	
Surgical resection	80 (66.7)
Percutaneous or endoscopic ultrasound-guided biopsy	40 (33.3)
Outcomes of surgical resection (n=80)	
Tumor location	
Hilar bile duct	32 (40.0)
Distal bile duct	48 (60.0)
R0 resection	47 (58.8)
R1 or R2 resection	33 (41.2)
Extent of tumor spread	
Right primary confluence invasion	18 (22.5)
Left primary confluence invasion	17 (21.3)
Right secondary confluence invasion	2 (2.5)
Left secondary confluence invasion	4 (5.0)
Hepatic invasion	12 (15.0)
Arterial invasion	6 (7.5)
PV invasion	10 (12.5)
Duodenal invasion	11 (13.8)
Pancreatic invasion	23 (28.8)
Regional lymph node metastasis	32 (40.0)

Among them, 80 underwent surgery as a study sample for diagnostic performance. Their location was pCCA:dCCA = 32:48 (40%:60%). Their histological type was mainly adenocarcinoma (adenocarcinoma = 75, mucinous carcinoma = 1, adenosquamous carcinoma = 1, adenocarcinoma with focal squamous differentiation = 2, and carcinoma in situ = 1). The outcomes of surgical resection are shown in Table 1.

Objective assessment

The mean ROI area of bile duct carcinoma in the portal phase was 174.0 mm^2 ^± 184.3 mm^2^ (mean±SD). The mean CT values of bile duct tumors and adjacent organs (abdominal aorta, portal vein, pancreas, and liver) in each phase are summarized in Table 2.

**Table 2:** CT values in the arterial, portal, and delayed phases of each anatomic location a, c, e) Bonferroni-adjusted p < 0.01 for the portal phase versus the arterial phase, p < 0.01 for the portal phase versus the delayed phase. b) Bonferroni-adjusted p < 0.01 for the arterial phase versus the portal phase, p < 0.01 for the arterial phase versus the delayed phase. d) Bonferroni-adjusted p < 0.01 for the arterial phase versus the delayed phase, p = 1 for the arterial phase versus the portal phase. Data are given as the mean CT value (Hounsfield units) ± standard deviation.

	Arterial phase	Portal phase	Delayed phase	p-value
Tumor	75.8 ± 9.3	102.3 ± 19.0	88.7 ± 14.6	<0.01^a^
Abdominal aorta	318.0 ± 53.2	167.7 ± 24.1	118.3 ± 15.2	<0.01^b^
Portal vein	108.1 ± 20.8	196.6 ± 29.3	119.3 ± 14.0	<0.01^c^
Pancreas	108.0 ± 33.1	107.0 ± 19.5	79.4 ± 16.4	<0.01^d^
Liver	71.0 ± 9.3	114.1 ±13.1	91.4 ± 16.4	<0.01^ e^

The bile duct tumor reached a peak contrast effect in the portal phase (99/120). Similarly, the liver and portal vein reached a peak contrast effect in the portal phase (liver: 119/120, portal vein: 116/120). The abdominal aorta and pancreas reached a peak contrast effect in the arterial phase (abdominal aorta: 120/120, pancreas: 62/120). Differences in CT values between adjacent organs and bile duct tumors in each phase are summarized in Table 3.

**Table 3:** CT value differences between adjacent organs and bile duct tumor in the arterial, portal, and delayed phases a, c) Bonferroni-adjusted p < 0.01 for the arterial phase versus the portal phase. Bonferroni-adjusted p < 0.01 for the arterial phase versus the delayed phase. b, d) Bonferroni-adjusted p < 0.01 for the portal phase versus the arterial phase. Bonferroni-adjusted p < 0.01 for the portal phase versus the delayed phase. Data are given as mean CT value (Hounsfield units) ± standard deviation.

	Arterial phase	Portal phase	Delayed phase	p
Abdominal aorta	241.8 ± 52.1	65.3 ± 23.2	29.3 ± 13.9	<0.01^ a^
Portal vein	32.4 ± 31.5	93.8 ± 25.5	30.4 ± 13.0	<0.01^b^
Pancreas	30.7 ± 25.7	5.1 ± 23.1	9.7 ± 18.7	<0.01^c^
Liver	5.1 ± 17.4	11.4 ± 16.6	2.3 ± 12.7	<0.01^d^

The portal vein and liver CT values showed the most difference from the bile duct tumor in the portal phase (p < 0.01). The abdominal aorta and pancreas CT values showed the most difference from the bile duct tumor in the arterial phase (p < 0.01).

Subjective assessment

The CT diagnostic performance in assessing the biliary segment-wise longitudinal tumor extent, arterial and PV invasion, organ invasion, and regional lymph node metastasis in each phase is summarized in Table 4.

**Table 4:** AUC and diagnostic performance for segmental and vascular invasion by extrahepatic cholangiocarcinoma ^a^ Values are presented as sensitivity, specificity, area under the curve (AUC) (95% confidence interval (CI)).

Parameter	Portal phase ^a^	Dual-phase image set ^a^	p	Triple-phase image set ^a^	p-value
Right primary confluence invasion					
Reader 1	0.72, 0.84, 0.77 (0.64–0.89)	0.72, 0.82, 0.76 (0.64–0.89)	1.00	0.72, 0.84, 0.77 (0.64–0.89)	0.88
Reader 2	0.72, 0.89, 0.82 (0.70–0.94)	0.72, 0.89, 0.82 (0.70–0.94)	0.52	0.72, 0.89, 0.81 (0.69–0.93)	0.18
Left primary confluence invasion					
Reader 1	0.77, 0.76, 0.78 (0.65–0.90)	0.88, 0.75, 0.83 (0.72–0.93)	0.15	0.88, 0.75, 0.84 (0.73–0.94)	0.09
Reader 2	0.82, 0.79, 0.83 (0.72–0.94)	0.82, 0.79, 0.84 (0.73–0.95)	0.14	0.88, 0.78, 0.85 (0.75–0.95)	0.42
Right secondary confluence invasion					
Reader 1	0.50, 0.90, 0.67 (0.17–1.00)	0.50, 0.90, 0.67 (0.16–1.00)	0.48	0.50, 0.92, 0.68 (0.13–1.00)	0.70
Reader 2	0.50, 0.89, 0.67 (0.18–1.00)	0.50, 0.89, 0.65 (0.15–1.00)	0.15	0.50, 0.91, 0.66 (0.13–1.00)	1.00
Left secondary confluence invasion					
Reader 1	0.75, 0.91, 0.82 (0.58–1.00)	0.75, 0.92, 0.82 (0.58–1.00)	0.32	0.75, 0.91, 0.80 (0.57–1.00)	0.33
Reader 2	0.75, 0.91, 0.82 (0.58–1.00)	0.75, 0.91, 0.81 (0.57–1.0)	0.36	0.75, 0.93, 0.82 (0.58–1.00)	0.79
Hilar bile duct invasion					
Reader 1	0.76, 0.91, 0.83 (0.75–0.92)	0.80, 0.91, 0.87 (0.79–0.95)	0.07	0.8, 0.88, 0.84 (0.75–0.93)	0.68
Reader 2	0.70, 0.94, 0.83 (0.75–0.92)	0.70, 0.94, 0.84 (0.75–0.92)	0.27	0.70, 0.94, 0.84 (0.76–0.92)	0.27
Distal bile duct invasion					
Reader 1	0.96, 0.65, 0.79 (0.65–0.91)	0.94, 0.65, 0.76 (0.63–0.89)	0.29	0.94, 0.65, 0.78 (0.66–0.90)	0.92
Reader 2	0.98, 0.62, 0.75 (0.62–0.88)	0.98, 0.62, 0.74 (0.61–0.88)	0.63	0.98, 0.62, 0.77 (0.65–0.89)	0.36
Hepatic invasion					
Reader 1	0.83, 0.85, 0.86 (0.73–098)	0.83, 0.87, 0.85 (0.73–0.98)	0.84	0.83, 0.88, 0.87 (0.75–0.99)	0.34
Reader 2	0.75, 0.87, 0.82 (0.68–0.96)	0.83, 0.88, 0.87 (0.75–1.00)	0.24	1.00, 0.75, 0.86 (0.72–1.00)	0.51
Pancreatic invasion					
Reader 1	0.83, 0.74, 0.79 (0.69–0.90)	0.83, 0.86, 0.87 (0.77–0.96)	0.01	0.91, 0.90, 0.93 (0.87–1.00)	<0.01
Reader 2	0.78, 0.90, 0.88 (0.79–0.96)	0.78, 0.95, 0.92 (0.85–1.00)	0.02	0.87, 0.97, 0.96 (0.91–1.00)	0.01
Duodenal invasion					
Reader 1	0.55, 0.86, 0.71 (0.55–0.88)	0.73, 0.90, 0.82 (0.67–0.96)	0.09	0.82, 0.90, 0.86 (0.74–0.99)	0.04
Reader 2	0.55, 0.91, 0.73 (0.57–0.89)	0.55, 0.93, 0.75 (0.59–0.91)	0.23	0.91, 0.93, 0.94 (0.85–1.00)	<0.01
PV invasion					
Reader 1	0.80, 0.91, 0.88 (0.74–1.00)	0.80, 0.91, 0.88 (0.74–1.00)	1.00	0.80, 0.91, 0.89 (0.74–1.00)	0.67
Reader 2	0.70, 0.90, 0.82 (0.66–0.97)	0.70, 0.90, 0.82 (0.66–0.97)	1.00	0.80, 0.97, 0.88 (0.74 –1.00)	0.21
Arterial invasion					
Reader 1	0.57, 0.95, 0.74 (0.51–0.96)	0.86, 1.00, 0.99 (0.96–1.00)	0.02	0.86, 1.00, 0.99 (0.96–1.00)	0.02
Reader 2	0.71, 0.82, 0.80 (0.60–1.00)	1.00, 0.89, 0.99 (0.97–1.00)	0.04	1.00, 0.89, 0.99 (0.97–1.00)	0.04
Regional lymph node metastasis					
Reader 1	0.84, 0.21, 0.52 (0.42–0.61)	0.84, 0.19, 0.51 (0.41–0.60)	0.32	0.84, 0.19, 0.51 (0.41–0.60)	0.32
Reader 2	0.94, 0.10, 0.50 (0.44–0.57)	0.91, 0.15, 0.51 (0.43–0.58)	0.78	0.88, 0.17, 0.48 (0.40–0.57)	0.57

The AUC of the bile duct segment in the portal phase was >0.7 as per both readers except for the right secondary confluence invasion. The sensitivity and specificity of the horizontal extension of CCA increased or decreased with the addition of arterial and delayed phases but with no statistically significant difference. Liver invasion and portal vein invasion also showed no improvement in diagnostic performance with the addition of imaging phases. Specificity for pancreatic invasion increased as per both readers when the arterial phase was added. Furthermore, both sensitivity and specificity increased when the delayed phase was added. The addition of the arterial phase increased sensitivity and specificity for duodenal invasion but with no statistically significant difference. However, adding the delayed phase significantly increased the sensitivity and AUC. Arterial invasion tended to be less sensitive in the portal phase, but sensitivity and specificity increased with the addition of the arterial phase. The addition of a delayed phase did not change the diagnostic performance.

**Figure 2:**
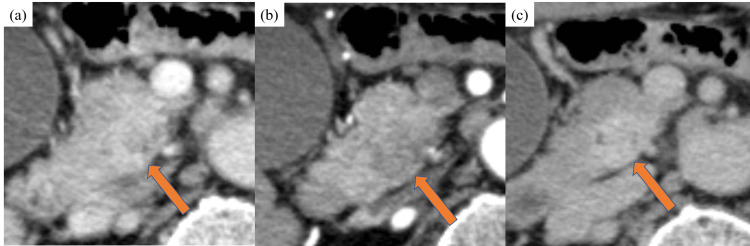
Contrast-enhanced CT images of a 73-year-old man with 26-mm distal bile duct cholangiocarcinoma (arrow) (a) Axial portal phase showing a contrast-enhanced mass with indistinct borders in the pancreatic head. (b) and (c) Axial arterial and delayed phases clearly showing a contrast-enhanced mass between the tumor and pancreatic parenchyma.

**Figure 3:**
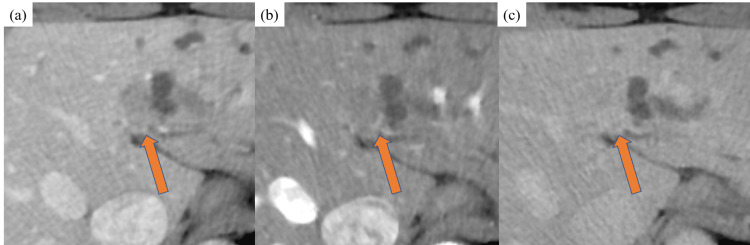
Contrast-enhanced CT images of a 74-year-old man with 20-mm perihilar bile duct cholangiocarcinoma (arrow) (a) Axial portal phase showing a low-density mass with the left primary and secondary bile duct; B2 and B3 bile ducts located upstream of the tumor are dilated. The mass extended beyond the bile duct and the parenchyma of the liver. (b) and (c) Axial arterial and delayed phases showing the mass demonstrated non-notable contrast enhancement compared with the liver parenchyma.

The sensitivity as per both readers for regional lymph node metastases in the portal phase tended to be high, but specificity was low. Diagnostic performance for regional lymph node metastases did not improve with the addition of the imaging phase. Representative cases are shown in Fig. 2 and Fig. 3.

Interobserver agreement was substantial to almost perfect (kappa = 0.71-0.93) except for the diagnosis of lymph node metastasis (Table 5).

**Table 5:** Interobserver agreement PV, portal vein. Data are presented as kappa (95% confidence interval (CI)).

Parameter	Portal phase	Dual-phase image set	Triple-phase image set
Right primary confluence invasion	0.86 (0.73, 0.98)	0.86 (0.75, 0.98)	0.90 (0.81, 0.99)
Left primary confluence invasion	0.84 (0.74, 0.95)	0.84 (0.73, 0.95)	0.84 (0.74, 0.94)
Right secondary confluence invasion	0.84 (0.71, 0.96)	0.86 80.74, 0.98)	0.90 (0.81, 1.00)
Left secondary confluence invasion	0.82 (0.66, 0.99)	0.91 (0.79, 1.03)	0.93 (0.85, 1.02)
Hilar bile duct invasion	0.91 (0.83, 0.98)	0.89 (0.81, 0.97)	0.87 (0.78, 0.95)
Distal bile duct invasion	0.87 (0.77, 0.97)	0.86 (0.75, 0.97)	0.88 (0.78, 0.98)
Hepatic invasion	0.92 (0.85, 0.98)	0.92 (0.87, 0.98)	0.77 (0.61, 0.93)
Pancreatic invasion	0.76 (0.64, 0.89)	0.80 (0.68, 0.92)	0.90 (0.84, 0.96)
Duodenal invasion	0.77 (0.61, 0.94)	0.71 (0.46, 0.95)	0.75 (0.56, 0.94)
PV invasion	0.79 (0.58, 0.99)	0.79 (0.58, 0.99)	0.88 (0.76, 0.99)
Arterial invasion	0.71(0.47, 0.94)	0.91 (0.80, 1.02)	0.91 (0.80, 1.02)
Regional lymph node metastasis	0.52 (0.29, 0.74)	0.47 (0.25, 0.70)	0.42 (0.21, 0.63)

## Discussion

The results of the objective assessment showed that the contrast effect of bile duct tumors was maximized in the portal phase. The contrast between bile duct tumors and arteries or the pancreas was maximized in the arterial phase. The subjective assessment results indicated an AUC of ≥0.7 in the one-phase image (portal phase only), excluding the right secondary confluence of the bile duct and lymph node metastasis. The addition of arterial and delayed phases significantly improved the AUC for arterial, pancreatic, and duodenal invasions, as observed by both readers (p < 0.05). There was no additional benefit regarding the horizontal spread of bile duct cancer, liver invasion, or portal vein invasion with the addition of imaging phases. These results suggest the importance of appropriate phase selection in multiphase CT imaging for diagnosing eCCA. Specifically, the information from the arterial and delayed phases on adding the portal phase helped evaluate pancreatic, duodenal, and arterial invasions. By contrast, the information from the portal phase alone was already sufficient for evaluating the segmental spread of bile ducts and liver and portal vein invasion.

eCCA tends to invade structures, such as the liver and bile ducts, but it can also invade other organs and blood vessels. The curative treatment for eCCA is surgical resection. Postoperative prognostic factors for eCCA include Bismuth classification, liver invasion, pancreatic invasion, duodenal invasion, lymph node metastasis, and achievement of R0 surgery, which significantly affect the five-year survival rate after surgery [10]. eCCA can involve organs, such as the liver, gallbladder, pancreas, duodenum, hepatic artery, and portal vein, each exhibiting characteristic contrast enhancement peaks [11]. Moreover, the administration of contrast agents and timing of scanning are crucial as inappropriate timing can result in unclear visualization of lesions that would otherwise be easily visible.

eCCA appears enhanced in the arterial and/or portal phases, while fibrotic components are better visualized in the delayed phase [12]. The portal phase is suitable for imaging the liver, incoming portal veins, and bile ducts, and it allows for obtaining the tumor’s characteristic contrast effect [13,14]. eCCA showed the highest contrast effectiveness in the portal phase. The high diagnostic accuracy observed in assessing the presence of eCCA and segmental extent of the bile duct during the portal phase emphasizes the significance of prioritizing portal-phase imaging for clinical diagnosis of eCCA. In addition, the sharp contrast between eCCA and the normal liver tissue in the portal phase potentially contributes to the detection of liver invasion. A CT value difference of at least 10 HU from the normal liver tissue is required for detecting liver lesions, which is consistent with the results of this study [15]. Conversely, hepatic invasion in the arterial and delayed phases did not exhibit a more pronounced contrast enhancement or a significant difference in CT values between hepatic invasion and eCCA, as compared to the portal phase. This suggests that the inclusion of arterial and delayed phases did not lead to an enhancement in diagnostic performance. The portal vein also demonstrated its highest contrast effect during the portal phase, highlighting the most distinguishable contrast difference from eCCA. Given the inherent contrast dynamics in these organs, the addition of arterial and delayed phases might not yield additional information regarding organ invasion beyond what the portal phase already provides.

Pancreatic, duodenal, and arterial invasions require accurate evaluation due to their impact on surgical planning and treatment strategies. The normal pancreatic parenchyma exhibits maximum enhancement in the arterial phase (pancreatic parenchymal phase) and gradually attenuates subsequently [11]. In pancreatic ductal carcinoma, enhancement gradually increases due to increased fibrous tissue, reaching its peak in the delayed phase [16]. Therefore, the difference in attenuation between pancreatic ductal carcinoma and the surrounding pancreatic parenchyma is maximized in the pancreatic parenchymal phase [17,18]. Fukukura et al. reported that by adding the delayed phase to consider pancreatic ductal carcinoma showing isodensity in the pancreatic parenchymal phase, the detection of pancreatic cancer showing isodensity in the pancreatic parenchymal phase was possible, leading to an improvement in the AUC [17]. In this study, the contrast between bile duct carcinoma and the pancreas was the highest in the arterial phase, followed by the delayed phase, as bile duct tumors reached the enhancement peak in the portal phase. Adding the arterial and delayed phases also improved the AUC for pancreatic invasion, suggesting that combining multiple phases is beneficial for diagnosing pancreatic invasion.

This study has several limitations, including its retrospective design and the fact that it was conducted at a single center. Furthermore, the relatively small number of patients included in the study highlights the necessity for larger-scale investigations. The imaging protocol used in this study, which encompasses contrast timing, slice thickness, and reconstruction method, was tailored to our institution, raising the possibility that these findings may not be replicable in other healthcare facilities. Notably, our study did not consider the potential impact of deep learning reconstruction (DLR) on tumor contrast enhancement, despite reports of its efficacy in other study [19]. In the context of imaging diagnosis for eCCA, it is essential to acknowledge the absence of a unified consensus. However, it is worth noting that recently published guidelines from the Korean Society of Abdominal Radiology stress the importance of multiphase CT imaging and specific slice thickness for CT imaging methods [12]. It is important to mention that this study did not employ multiplanar reconstruction (MPR) or curved planar reconstruction (CPR) in the reading experiment [20,21]. While MPR and CPR have previously been documented as useful for assessing the horizontal spread of bile duct tumors, the absence of these techniques in our study may have led to an underestimation of the sensitivity, particularly in detecting the horizontal spread of the right secondary confluence. Furthermore, this study did not evaluate precontrast imaging, which could potentially impact post-contrast results, mainly due to the technical challenge of placing ROIs on the tumor prior to contrast administration. The relative inadequacy of specificity in diagnosing lymph node metastasis in this study, even with the incorporation of arterial and delayed phases, indicates the need for further research to improve diagnostic performance in this aspect. In addition, the interobserver agreement for assessing lymph node metastasis was comparatively lower than that for other organs, suggesting that the criteria employed in this study to evaluate lymph node metastasis were unique and might have influenced the diagnostic performance.

## Conclusions

This study elucidated the role of multiphase CT in diagnosing eCCA and demonstrated that appropriate phase selection contributes to improved diagnostic performance. Specifically, the portal-phase information is sufficient for evaluating the segmental extent of bile duct, liver, and portal vein invasion. Information from the arterial and delayed phases is valuable for assessing pancreatic, duodenal, and arterial invasions.

## References

[1] Nagtegaal ID, Odze RD, Klimstra D (2020). The 2019 WHO classification of tumours of the digestive system. Histopathology.

[2] Banales JM, Marin JJ, Lamarca A (2020). Cholangiocarcinoma 2020: the next horizon in mechanisms and management. Nat Rev Gastroenterol Hepatol.

[3] Elvevi A, Laffusa A, Scaravaglio M (2022). Clinical treatment of cholangiocarcinoma: an updated comprehensive review. Ann Hepatol.

[4] Kovalenko YA, Zharikov YO, Konchina NA, Gurmikov BN, Marinova LA, Zhao AV (2020). Perihilar cholangiocarcinoma: a different concept for radical resection. Surg Oncol.

[5] Yu Z, Zhu J, Jiang H, He C, Xiao Z, Wang J, Xu J (2018). Surgical resection and prognostic analysis of 142 cases of hilar cholangiocarcinoma. Indian J Surg.

[6] Kwon HJ, Kim SG, Chun JM, Lee WK, Hwang YJ (2014). Prognostic factors in patients with middle and distal bile duct cancers. World J Gastroenterol.

[7] Ni Q, Wang H, Zhang Y (2017). MDCT assessment of resectability in hilar cholangiocarcinoma. Abdom Radiol (NY).

[8] (2022). NCCN clinical practice guidelines in oncology (NCCN Guidelines®) hepatobiliary cancers (version 1.2022). https://www.nccn.org/guidelines/guidelines-process/transparency-process-and-recommendations/GetFileFromFileManagerGuid?FileManagerGuidId=98491994-dc79-495d-9166-8c3fa3315adc.

[9] (2022). NCCN clinical practice guidelines in oncology (NCCN Guidelines®) pancreatic adenocarcinoma (version 1.2022). https://www.nccn.org/guidelines/guidelines-process/transparency-process-and-recommendations/GetFileFromFileManagerGuid?FileManagerGuidId=e63411af-32dd-42a0-a9fc-9bf5babb67d2.

[10] Murakami Y, Uemura K, Hayashidani Y, Sudo T, Ohge H, Sueda T (2007). Pancreatoduodenectomy for distal cholangiocarcinoma: prognostic impact of lymph node metastasis. World J Surg.

[11] Goshima S, Kanematsu M, Kondo H (2006). Pancreas: optimal scan delay for contrast-enhanced multi-detector row CT. Radiology.

[12] Lee DH, Kim B, Lee ES (2021). Radiologic evaluation and structured reporting form for extrahepatic bile duct cancer: 2019 Consensus recommendations from the Korean Society of Abdominal Radiology. Korean J Radiol.

[13] Choi SH, Han JK, Lee JM, Lee KH, Kim SH, Lee JY, Choi BI (2005). Differentiating malignant from benign common bile duct stricture with multiphasic helical CT. Radiology.

[14] Choi YH, Lee JM, Lee JY, Han CJ, Choi JY, Han JK, Choi BI (2008). Biliary malignancy: value of arterial, pancreatic, and hepatic phase imaging with multidetector-row computed tomography. J Comput Assist Tomogr.

[15] Baron RL (1994). Understanding and optimizing use of contrast material for CT of the liver. AJR Am J Roentgenol.

[16] Scialpi M, Cagini L, Pierotti L (2014). Detection of small (≤ 2 cm) pancreatic adenocarcinoma and surrounding parenchyma: correlations between enhancement patterns at triphasic MDCT and histologic features. BMC Gastroenterol.

[17] Fukukura Y, Kumagae Y, Fujisaki Y (2021). Adding delayed phase images to dual-phase contrast-enhanced CT increases sensitivity for small pancreatic ductal adenocarcinoma. AJR Am J Roentgenol.

[18] Fletcher JG, Wiersema MJ, Farrell MA (2003). Pancreatic malignancy: value of arterial, pancreatic, and hepatic phase imaging with multi-detector row CT. Radiology.

[19] Tamura A, Mukaida E, Ota Y, Nakamura I, Arakita K, Yoshioka K (2022). Deep learning reconstruction allows low-dose imaging while maintaining image quality: comparison of deep learning reconstruction and hybrid iterative reconstruction in contrast-enhanced abdominal CT. Quant Imaging Med Surg.

[20] Nagakawa Y, Kasuya K, Bunso K (2014). Usefulness of multi-3-dimensional computed tomograms fused with multiplanar reconstruction images and peroral cholangioscopy findings in hilar cholangiocarcinoma. J Hepatobiliary Pancreat Sci.

[21] Kakihara D, Yoshimitsu K, Irie H (2007). Usefulness of the long-axis and short-axis reformatted images of multidetector-row CT in evaluating T-factor of the surgically resected pancreaticobiliary malignancies. Eur J Radiol.

